# Prognostic significance of CXCL5 expression in cancer patients: a meta-analysis

**DOI:** 10.1186/s12935-018-0562-7

**Published:** 2018-05-02

**Authors:** Binwu Hu, Huiqian Fan, Xiao Lv, Songfeng Chen, Zengwu Shao

**Affiliations:** 1 Department of Orthopaedics, Union Hospital, Tongji Medical College, Huazhong University of Science and Technology, Wuhan, 430022 China; 2 Division of Gastroenterology, Union Hospital, Tongji Medical College, Huazhong University of Science and Technology, Wuhan, 430022 China; 3 Department of Orthopaedic Surgery, The First Affiliated Hospital of Zhengzhou University, Zhengzhou, China

**Keywords:** Chemokine, CXCL5, Cancer, Prognosis, Meta-analysis

## Abstract

**Background:**

CXCL5 is a member of the CXC-type chemokine family, which has been found to play important roles in tumorigenesis and cancer progression. Recent studies have demonstrated that CXCL5 could serve as a potential prognostic biomarker for cancer patients. However, the prognostic value of CXCL5 is still controversial.

**Methods:**

We systematically searched PubMed, Embase and Web of Science to obtain all relevant articles investigating the prognostic significance of CXCL5 expression in cancer patients. Hazards ratios (HR) with corresponding 95% confidence intervals (CI) were pooled to estimate the association between CXCL5 expression levels with survival of cancer patients.

**Results:**

A total of 15 eligible studies including 19 cohorts and 5070 patients were enrolled in the current meta-analysis. Our results demonstrated that elevated expression level of CXCL5 was significantly associated with poor overall survival (OS) (pooled HR 1.70; 95% CI 1.36–2.12), progression-free survival (pooled HR 1.65; 95% CI 1.09–2.49) and recurrence-free survival (pooled HR 1.49; 95% CI 1.15–1.93) in cancer patients. However, high or low expression of CXCL5 made no difference in predicting the disease-free survival (pooled HR 0.63; 95% CI 0.11–3.49) of cancer patients. Furthermore, we found that high CXCL5 expression was associated with reduced OS in intrahepatic cholangiocarcinoma (HR 1.91; 95% CI 1.31–2.78) and hepatocellular carcinoma (HR 1.87; 95% CI 1.55–2.27). However, there was no significant association between expression level of CXCL5 with the OS in lung cancer (HR 1.25; 95% CI 0.79–1.99) and colorectal cancer (HR 1.16; 95% CI 0.32–4.22, p = 0.826) in current meta-analysis.

**Conclusions:**

In conclusion, our meta-analysis suggested that elevated CXCL5 expression might be an adverse prognostic marker for cancer patients, which could help the clinical decision making process.

## Background

Despite great improvements in early detection, surgical techniques, chemotherapy, radiotherapy, biological treatment and multidisciplinary treatment in recent years, cancer is still a major public health problem globally, which is associated with high morbidity, mortality and economic burden [1]. It is estimated that 1,735,350 new cancer cases and 609,640 cancer deaths are projected to occur in the United States in 2018 [2]. Given the poor prognosis of cancer patients, numerous investigators have focused on searching for biomarkers that could predict prognosis of cancer. However, sensitivity and specificity of most cancer biomarkers widely used now are not yet satisfactory [3]. Therefore, it is desperately needed to identify novel applicable prognostic biomarkers, not only improving poor prognosis but also providing novel therapeutic targets.

Chemokines are chemotactic cytokines that could regulate the migration of immune cells into damaged or diseased organs in response to pro-inflammatory stimuli [4]. According to cysteine residues in the NH2-terminal part of the protein, chemokines can be classified into four highly conserved groups, namely C, CC, CXC, and CX3C [5]. Chemokines and their receptors could bring about the transcription of target genes involved in cell invasion, motility, survival and interactions with the extracellular matrix, which can induce migration, chemotaxis and rearrangement of the cytoskeleton in the target cell, and therefore promote multiple physiological functions of cells, including cell growth, development, differentiation and apoptosis [6–9]. Over the past few years, accumulating evidence has revealed that chemokines play pivotal roles in progression of tumor [10]. Chemokines produced by tumor and stromal cells can induce the expression and distribution of tumor-associated leukocytes, trigger angiogenesis, contribute to the growth and metastasis of malignant cells and generate fiber keratinocytes [6, 11, 12]. In addition, chemokines and their receptors are critical mediators of inflammation microenvironment of cancer, which has been proposed to represent the seventh hallmark of cancer [13, 14]. Given the important roles of chemokines in cancer, abnormal expression of chemokines has been detected in many tumors, and several chemokines have been proven to be associated with poor prognosis of cancer patients [15–17].

CXCL5, also known as epithelial-derived neutrophil-activating peptide 78 (ENA78), is originally discovered as a potent chemoattractant and activator of neutrophil function. Through binding to its receptor CXCR2, CXCL5 could induce the chemotaxis of neutrophils, promote angiogenesis, and remodel connective tissue [18]. Accumulating evidence suggests that CXCL5 may participate in cancer-related inflammation, which is involved in many aspects of malignancy in cancer biology [19]. Furthermore, abnormal expression of CXCL5 has been identified in many tumors. CXCL5 is overexpressed in gastric cancer, prostate cancer, endometrial cancer, squamous cell cancer, hepatocellular carcinoma and pancreatic cancer, and increased expression of CXCL5 is associated with advanced tumor stages, local invasion, neutrophil infiltration and metastatic potential [20–25]. Recent studies have revealed that CXCL5 could serve as a potential prognostic biomarker for patients with cancer [5, 19, 26, 27]. However, its prognostic value is still controversial owing to the fact that most studies reported so far are limited in discrete outcome and sample size. Therefore, we performed the current quantitative meta-analysis to elucidate the prognostic significance of CXCL5 expression in cancer patients.

## Materials and methods

### Study strategy

The present review was performed in accordance with the standard guidelines for meta-analysis and systematic reviews of tumor marker prognostic studies [28, 29]. The database Web of Science, PubMed and Embase were independently searched by two researchers (Binwu Hu and Huiqian Fan) to obtain all relevant articles about the prognostic value of CXCL5 in patients with any tumor. The literature search ended on March 1, 2018. The search strategy used both MeSH terminology and free-text words to increase the sensitivity of the search. The search strategy was: “CXCL5 or CXC chemokine ligand 5 or ENA78 or epithelial cell derived neutrophil attractant 78” AND “cancer or tumor or carcinoma or neoplasm or malignancy” AND “prognostic or prognosis or survival or outcome”. We also screened the references of retrieved relevant articles to identify potentially eligible literatures. Conflicts were solved through group discussion.

### Inclusion and exclusion criteria

Studies included in this analysis had to meet the following inclusion criteria: (1) patients were pathologically diagnosed with any type of human cancer. (2) CXCL5 expression levels were determined in human tissues or plasma samples. (3) Patients were divided into two groups according to the expression levels of CXCL5, the relationship between CXCL5 expression levels with survival outcome was investigated. (4) Sufficient published data or the survival curve were provided to calculate hazard ratios (HR) for survival rates and their 95% confidence intervals (CI). Exclusion criteria were as follow: studies using non-human samples, studies without usable or sufficient data, laboratory articles, reviews, letters, case reports, non-English or unpublished articles and conference abstracts. All eligible studies were carefully screened by two researchers (Binwu Hu and Huiqian Fan), and discrepancies were resolved by discussing with a third researcher (Xiao Lv).

### Data extraction

Two investigators (Binwu Hu and Huiqian Fan) extracted relevant data independently and reached a consensus on all items. For all eligible studies, the following information of each article was collected: author, year of publication, tumor type, samples detected, expression associated with poor prognosis, Newcastle–Ottawa Scale (NOS) score, method of obtaining HRs, characteristics of the study population (including country of the population enrolled, number of patients (high/low), follow up (month)), endpoints, assay method, cut-off value and survival analysis. For endpoints, overall survival (OS), disease-free survival (DFS), progression-free survival (PFS) and recurrence-free survival (RFS) were all regarded as endpoints. We employed HR which was extracted following a methodology suggested previously to evaluate the influence of CXCL5 expression on prognosis of patients [30]. If possible, we also asked for original data directly from the authors of the relevant studies.

### Quality assessment

Quality of all included studies was assessed independently by two researchers (Binwu Hu and Huiqian Fan) using the validated Newcastle–Ottawa Scale, and disagreements were resolved through discussion with another researcher (Songfeng Chen). This scale uses a star system to evaluate a study in three domains: selection of participants, comparability of study groups, and the ascertainment of outcomes of interest. We considered studies with scores more than 6 as high-quality studies, and those with scores no more than 6 as low-quality studies.

### Statistical analysis

Statistical analysis was performed using Stata Software 14.0 (Stata, College Station, TX). Pooled HRs (high/low) and their associated 95% CIs were used to analyze the prognostic role of CXCL5 expression in various cancers. The heterogeneity among studies was evaluated using Cochran’s Q and I^2^ statistics. A p value less than 0.10 or an I^2^ value larger than 50% were considered statistically significant. The fixed-effect model was used for analysis without significant heterogeneity between studies (p > 0.10, I^2^ < 50%). Otherwise, the random-effect model was chosen. To explore the source of heterogeneity, subgroup analysis and meta-regression were preformed through classifying the included studies into subgroups according to similar features. We also conducted sensitivity analysis to test the effect of each study on the overall pooled results. The publication bias was evaluated by using both Begg’s test and Egger’s test. A p value less than 0.05 was considered statistically significant.

## Results

### Characteristics of studies

**Fig. 1:**
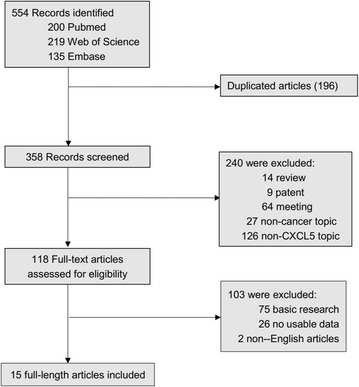
The flow diagram indicated the process of study selection

According to our search strategy, the initial search algorithm retrieved a total of 554 studies. The following studies were excluded: duplicates (n = 196), review (n = 14), patent (n = 9), meeting abstract (n = 64), studies describing non-cancer topics (n = 27), studies describing non-CXCL5 topics (n = 126), studies belonging to basic research (n = 75), studies lacking relevant data (n = 26) and non-English articles (n = 2). Eventually, 15 studies meeting the inclusion criteria were included in this meta-analysis. The screening process and results are shown in Fig. 1.

The main characteristics of the included studies are shown in Table 1. A total of 15 studies including 19 cohorts were included in the current meta-analysis. Among these studies, a total of 5070 patients were included, with a minimum sample size of 27 and a maximum sample size of 2437 patients. The accrual period of these studies ranged from 2007 to 2018. The follow-up time ranged from 23 months to 180 months. Ten different types of cancer were involved in the enrolled studies including intrahepatic cholangiocarcinoma (n = 2) [27, 31], lung cancer (n = 3) [26, 32, 33], colorectal cancer (n = 3) [34–36], biliary tract cancer (n = 1) [10], breast cancer (n = 1) [5], bladder cancer (n = 1) [19], glioma (n = 1) [18], pancreatic cancer (n = 1) [25], hepatocellular carcinoma (n = 1) [24] and nasopharyngeal carcinoma (n = 1) [37]. Among these studies, OS (n = 14), DFS (n = 3), PFS (n = 3) and RFS (n = 3) were estimated as survival outcome. The CXCL5 expression levels in these studies were mostly measured by using immunohistochemistry (IHC) technique, while real time PCR (RT-PCR) and enzyme-linked immunosorbent assay (ELISA) were also applied. Because the cut-off definitions were various, the cut-off values were different in these studies.

**Table 1:** Characteristics of studies included in the meta-analysis

Author	Year	Region	Type of cancer	Sample size (high/low)	Follow-up (month)	Endpoints	Expression associated with poor prognosis	Samples detected	Assay method	Cut-off value	Survival analysis	NOS score	Method
Lee et al.	2018	Korea	Biliary tract cancer	4/23	23	OS	High	Blood	ELISA	High: serum CXCL5 levels were > 2.081 ng/mL	Multivariate	7	1
Bièche et al.	2007	France	Breast cancer	24/24	120	RFS	High	Tissue	RT-PCR	ROC	Univariate	7	2
Oksana et al.	2014	Poland	Lung cancer	37/37	82	OS, DFS	Low	Tissue	RT-PCR	High: the gene expression of CXCL5 in tumor tissue was more than 1.08 times than normal tissue	Multivariate	7	2
Zhou et al.	2014	China	Intrahepatic cholangiocarcinoma	70/70	120	OS	High	Tissue	IHC	Median	Univariate Multivariate	7	1
Zhu et al.	2016	China	Bladder cancer	131/124	87	OS, RFS, PFS	High	Tissue	IHC	High: IRS > 4	NA	7	2
Dai et al.	2016	China	Glioma	34/31	48	OS	High	Tissue	WB, RT-PCR	Median	NA	6	2
Wu et al.	2017	China	Lung cancer	75	60	OS	High	Tissue	IHC,RT-PCR	High: the multiplication for intensity and proportion was more than 2	Univariate Multivariate	7	2
				2437	60	OS, PFS	High	Tissue	RT-PCR	Median	Univariate	6	1
Kawamura et al.	2011	Japan	Colorectal cancer	69/181	104	OS	High	Blood	ELISA	High: serum CXCL5 levels were > 1.53 ng/mL	Univariate Multivariate	7	1
Han et al.	2015	China	Lung adenocarcinoma	34/192	127	OS,RFS	High	Tissue	RT-PCR	Median	Univariate	6	2
Speetjens et al.	2008	The Netherlands	Colorectal cancer	Cohort 1 53/17	172	DFS	Low	Tissue	RT-PCR	The 25th percentile as cut off point	Univariate Multivariate	7	1
				Cohort 2 50/8	162	OS	Low	Tissue	IHC	High: CXCL5 expression in > 50% of the tumor cells	Univariate Multivariate	7	1
Okabe et al.	2012	Japan	Intrahepatic cholangiocarcinoma	25/25	126.3	OS	High	Tissue	IHC	High: a percentage of the total number of stained cells > 10%	NA	6	2
Li et al.	2010	USA	Pancreatic cancer	130/23	180	OS	High	Tissue	IHC	High: percentage of tumor cells staining positively for CXCL5 > 5.5%	NA	6	2
Zhou et al.	2012	China	Hepatocellular carcinoma	47/47	50	OS	High	Tissue	IHC	Median	Univariate Multivariate	8	1
				162/161	75	OS	High	Tissue	IHC	Median	Univariate Multivariate	8	1
				251/251	75	OS	High	Tissue	IHC	Median	Univariate Multivariate	8	1
Zhang et al.	2013	China	Nasopharyngeal carcinoma	75/70	105	OS, PFS	High		ELISA	High: serum CXCL5 levels were > 0.805 ng/ml	Univariate Multivariate	7	1
Zhao et al.	2017	China	Colorectal cancer	48/30	60	OS, DFS	High		IHC	High: a staining score of 4.5 as the cut-off value	Univariate Multivariate	8	1

Method: 1 denoted as obtaining HRs directly from publications; 2 denoted as HRs calculated from the total number of events, corresponding p value and data from Kaplan–Meier curves

*OS* overall survival, *DFS* disease-free survival, *PFS* progression-free survival, *RFS* recurrence-free survival, *IHC* immunohistochemistry, *RT*-*PCR* real time polymerase chain reaction, *ELISA* enzyme-linked immunosorbent assay, *NA* not available, *ROC* receiver operating characteristics, *NOS* Newcastle–Ottawa Scale

### Association between CXCL5 expression levels with OS of cancer patients

**Fig. 2:**
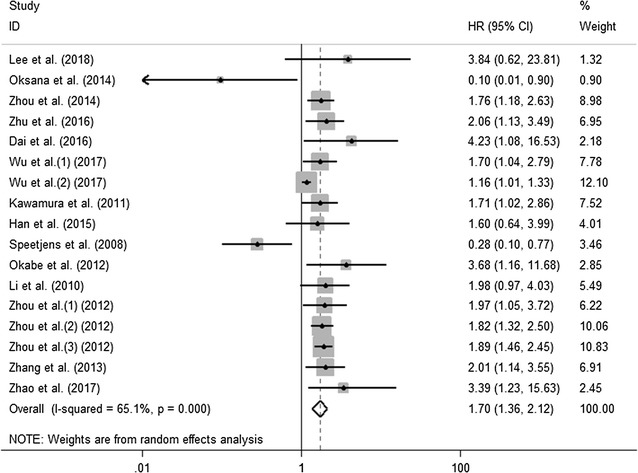
Meta-analysis of the pooled HRs of OS for cancer patients

**Fig. 3:**
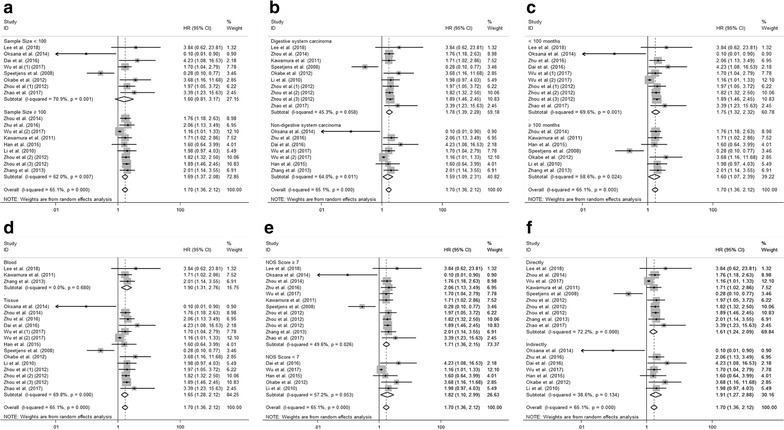
Results of subgroup analysis of pooled HRs of OS for cancer patients. **a** Subgroup analysis stratified by sample size. **b** Subgroup analysis stratified by type of cancer. **c** Subgroup analysis stratified by follow-up time. **d** Subgroup analysis stratified by sample detected. **e** Subgroup analysis stratified by NOS score. **f** Subgroup analysis stratified by source of HR

Fourteen studies including seventeen cohorts reported the relationship between abnormal expression levels of CXCL5 with OS in a total of 4952 cancer patients. We used random-effect model to calculate the pooled HR. The pooled HR for OS was 1.70 (95% CI 1.36–2.12, p < 0.001), which suggested that elevated expression level of CXCL5 was significantly associated with poor OS in cancer patients (Fig. 2). Given that significant heterogeneity existed among studies (I^2^ = 65.1%; p < 0.001), we further conducted subgroup analysis by factors of sample size (fewer than 100 or more than 100), type of cancer (digestive system or non-digestive system carcinoma), follow-up time (fewer than 100 or more than 100 months), samples detected (blood or tissue), paper quality (NOS scores ≥ 7 or < 7) and source of HR (directly or indirectly) to explore the source of heterogeneity (Fig. 3a–f). The results of subgroup analysis illustrated that the association between increased expression level of CXCL5 with poor OS of cancer patients was still significant in all factors above except for the subgroup of studies with fewer than 100 patients (HR 1.60, 95% CI 0.81–3.17, p = 0.175) (Table 2). To further explore the sources of heterogeneity, we performed meta-regression by the covariates including above factors. However, meta-regression didn’t reveal p values less than 0.05 in above covariates, which indicated that all above factors were not the sources of heterogeneity (Table 2). Furthermore, using Cox multivariate analysis in eight studies including ten cohorts, we found that elevated CXCL5 expression levels was an independent prognostic factor for OS in cancer patients (HR 1.65, 95% CI 1.24–2.20, p = 0.001).

**Table 2:** Subgroup analysis of pooled HRs for OS in cancer patients with abnormal expression level of CXCL5

Subgroup analysis	No. of cohorts	Pooled HRs	Meta regression (p value)	Heterogeneity	
		Random		I^2^ (%)	p value
Sample size			0.602		
< 100	8	1.60 [0.81–3.17]	–	70.9	0.001
≥ 100	9	1.69 [1.37–2.08]	–	62.0	0.007
Type of cancer			0.197		
Digestive system carcinoma	10	1.78 [1.39–2.28]	–	45.3	0.058
Non-digestive system carcinoma	7	1.59 [1.09–2.31]	–	64.0	0.011
Follow-up time			0.204		
< 100	10	1.75 [1.32–2.32]	–	69.6	0.001
≥ 100	7	1.60 [1.07–2.39]	–	58.6	0.024
Samples detected			0.186		
Blood	3	1.90 [1.31–2.76]	–	0.0	0.680
Tissue	14	1.65 [1.28–2.12]	–	69.8	0.000
NOS score			0.526		
≥ 7	12	1.71 [1.36–2.15]	–	49.6	0.026
< 7	5	1.82 [1.10–2.99]	–	57.2	0.053
Source of HR			0.209		
Directly	10	1.61 [1.24–2.09]	–	72.2	0.000
Indirectly	7	1.91 [1.27–2.88]	–	38.6	0.134

### Association between CXCL5 expression levels with OS of certain types of cancer

**Fig. 4:**
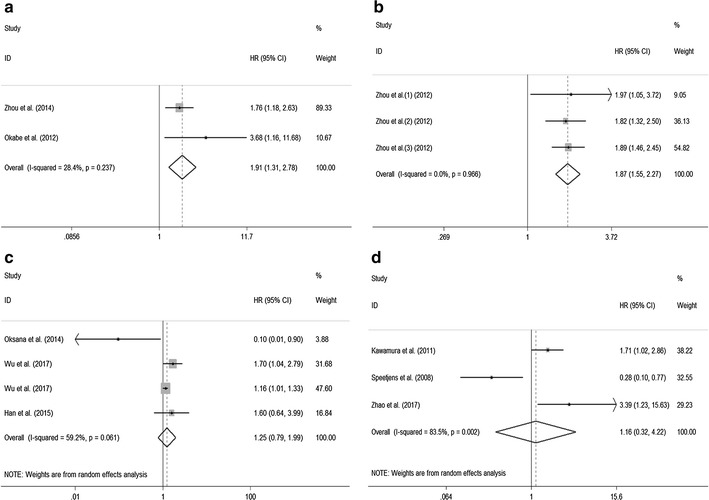
Meta-analysis of the pooled HRs of OS for intrahepatic cholangiocarcinoma (**a**), hepatocellular carcinoma (**b**), lung cancer (**c**), and colorectal cancer (**d**)

We further evaluated the prognostic value of CXCL5 in certain types of cancer. Through systematic analysis, our results demonstrated that high CXCL5 expression was associated with reduced OS in intrahepatic cholangiocarcinoma (HR 1.91; 95% CI 1.31–2.78, p = 0.001) (Fig. 4a) and hepatocellular carcinoma (HR 1.87; 95% CI 1.55–2.27, p < 0.001) (Fig. 4b). However, there was no significant association between expression level of CXCL5 with OS of cancer patients in lung cancer (HR 1.25; 95% CI 0.79–1.99, p = 0.335) (Fig. 4c) and colorectal cancer (HR 1.16; 95% CI 0.32–4.22, p = 0.826) (Fig. 4d).

### Association between CXCL5 expression levels with DFS, PFS and RFS of cancer patients

**Fig. 5:**
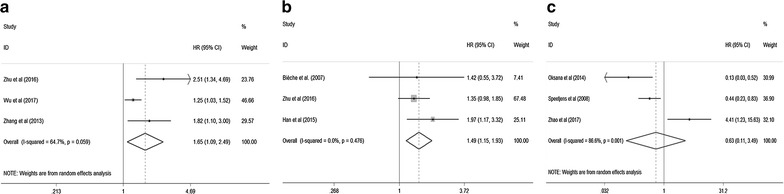
Meta-analysis of the pooled HRs of PFS (**a**), RFS (**b**) and DFS (**c**) for cancer patients

There were three studies respectively evaluating the relationship between CXCL5 expression levels with DFS, PFS and RFS. Through systematic analysis, our results revealed that higher expression level of CXCL5 was significantly associated with shorter PFS (HR 1.65; 95% CI 1.09–2.49, p = 0.018) (Fig. 5a) and RFS (HR 1.49; 95% CI 1.15–1.93, p = 0.003) (Fig. 5b). However, high or low expression of CXCL5 made no difference in predicting the DFS (HR 0.63; 95% CI 0.11–3.49, p = 0.595) (Fig. 5c). In addition, due to the limited number of included studies, we did not perform the subgroup analysis.

### Sensitivity analysis and publication bias

**Fig. 6:**
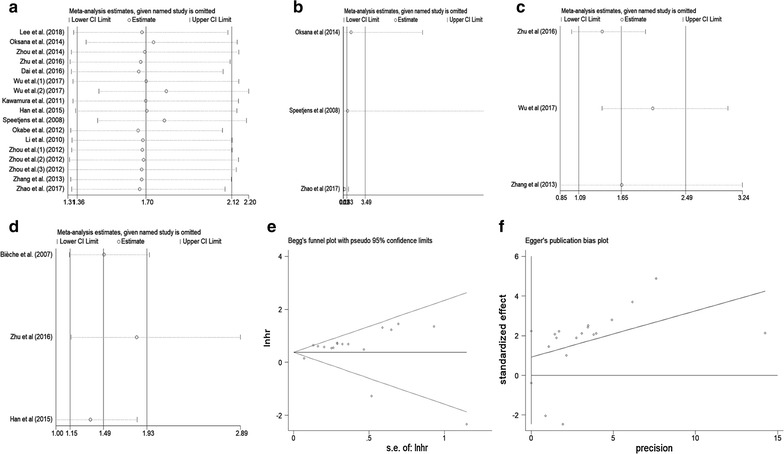
Sensitivity analysis plot of pooled HRs of OS (**a**), DFS (**b**), PFS (**c**) and RFS (**d**) for cancer patients with abnormally expressed CXCL5. Begg’s test (**e**) and Egger’s test (**f**) for publication bias

We performed sensitivity analysis to examine the effects of individual study on the overall results. For OS, the sensitivity analysis identified that results from Wu et al. (2) and Speetjens et al. affected results greatly, which indicated that these two studies were possible to be the main source of heterogeneity. However, the list of pooled HRs and 95% CIs after excluding single study one by one indicated robustness of our results, in which all pooled HRs and 95% CIs were above the null hypothesis of 1 (Fig. 6a). For DFS (Fig. 6b) and PFS (Fig. 6c), the sensitivity analysis revealed that all included studies affected results greatly. For RFS, only the results from Bièche et al. did not influence the results greatly (Fig. 6d). The sensitivity analysis results demonstrated that our results for DFS, PFS and RFS were not that stable, which might be because of the limited number of studies included in each analysis. Therefore, more relevant studies are warranted to investigate the effects of CXCL5 on DFS, PFS and RFS in human cancer.

Begg’s test and Egger’s linear regression test were conducted to evaluate publication bias. For OS, Begg’s test (p = 0.773) (Fig. 6e) and Egger’s test (p = 0.157) (Fig. 6f) showed no significant publication bias across studies. For DFS, PFS and RFS, because of the limited number of studies (below 10) included in each analysis, publication bias was not assessed.

## Discussion

CXCL5 is originally discovered as a potent chemoattractant and activator of neutrophil function [33]. Through interacting with CXCR2 receptor, it could function both as a chemoattractant and as an angiogenic factor [35, 38, 39]. Recently, CXCL5 has been shown to be able to promote the proliferation, migration and invasion of various tumor cells and play pivotal roles in the pathogenesis and progression of cancer [27, 37]. It was reported that CXCL5 protein was higher in various lung cancer tissues, which was positively associated with tumor stage, lymph node metastasis, and worse survival [26] [19, 40]. Zhou et al. also reported that CXCL5 was overexpressed in intrahepatic cholangiocarcinoma cell lines and tumor samples, which could promote intrahepatic cholangiocarcinoma growth and metastasis by recruiting intratumoral neutrophils [27]. Furthermore, CXCL5 could directly induce endothelial cell proliferation and invasion in vitro and promote tumor angiogenesis in non-small cell lung carcinoma and pancreatic cancer [41–43]. Considering the important functions of CXCL5 in cancer, studies have demonstrated that CXCL5 could serve as a potential prognostic biomarker for cancer patients. However, the prognostic value of CXCL5 is still controversial. Because even in the same type of tumor, there are almost opposite conclusions about the prognostic value of CXCL5 [34–36].

Here we performed the current comprehensive meta-analysis to systematically explore the prognostic value of abnormally expressed CXCL5 in cancer patients. We examined 15 independent studies including 19 cohorts and 5070 patients. Through systematic analysis, our results demonstrated that high expression level of CXCL5 was significantly associated with poor OS in cancer patients. Due to the significant heterogeneity across these studies, we performed subgroup analysis and meta-regression analysis to explore the sources of heterogeneity. The results of subgroup analysis suggested that sample size (fewer than 100 or more than 100) altered the significance of prognostic role of CXCL5 in OS (HR 1.60, 95% CI 0.81–3.17 vs HR 1.69, 95% CI 1.37–2.08). This indicated that difference in sample size might be the source of heterogeneity. However, meta-regression analysis failed to identify the source of the significant heterogeneity in above covariates. In addition, by combining HRs from Cox multivariate analysis, we found that CXCL5 was an independent prognostic factor of OS in cancer patients.

Furthermore, we evaluated the prognostic value of CXCL5 in certain types of cancer. We found that high CXCL5 expression was associated with reduced OS in intrahepatic cholangiocarcinoma and hepatocellular carcinoma, which was consistent with previous studies. However, there was no significant association between expression level of CXCL5 with the OS of lung cancer and colorectal cancer. For lung cancer, results from Oksana et al. were contrary to others greatly [32]. The reason might be that they only evaluated the prognostic value of CXCL5 in early stage non-small cell lung cancer (stages I and II) [32]. Similarly, for colorectal cancer, the results from Speetjens et al. also conflicted with others because they did not include stage IV patients [35, 36]. Therefore, we may speculate that CXCL5 might have different prognostic roles in different tumor stage and larger-scale, multicenter studies including all stage patients are needed to verify our hypothesis.

DFS, PFS and RFS are all important parameters reflecting the progression of tumor. Our results demonstrated that higher expression level of CXCL5 was significantly associated with shorter PFS and RFS in cancer patients. However, high or low expression of CXCL5 made no difference in predicting the DFS of cancer patients. In addition, because only three studies respectively were included to evaluate the association between CXCL5 expression levels with DFS, PFS and RFS, more studies are necessary to explore the relationship between CXCL5 with tumor progression.

Mechanisms underlying the regulatory role of CXCL5 in tumorigenesis and tumor progression have been extensively investigated. CXCL5 could activate multiple signaling pathways to promote the progression of cancer. Dai et al. found that overexpression of CXCL5 markedly upregulated the activity of the JNK, ERK and p38 MAPK signaling pathways, which may contribute to the promoting effects of CXCL5 on the proliferation and migration of glioma cells [18]. In bladder cancer, CXCL5 was found to be significantly upregulated and the CXCL5/CXCR2 axis could promote the migration and invasion of bladder cancer cells by activating the PI3K/AKT-induced upregulation of MMP2/MMP9 [19, 40]. The CXCR2/CXCL5 axis was also found to enhance epithelial-mesenchymal transition of hepatocellular carcinoma cells through the activation of the PI3K/AKT/GSK-3β/Snail signaling [44]. Furthermore, Hsu et al. demonstrated that progression of breast cancer induced by TAOB-derived CXCL5 was associated with increased Raf/MEK/ERK activation and mitogen- and stress-activated protein kinase 1 (MSK1) and Elk-1 phosphorylation, as well as Snail upregulation [44]. In addition, CXCL5 was shown to have potent effects on neutrophil recruitment in cancer [45, 46]. Meanwhile, neutrophils could potentiate cancer cell migration, invasion and dissemination by secreting immunoreactive molecules such as hepatocyte growth factor, oncostatin M, b2-integrins or neutrophil elastase, which might be another mechanism for CXCL5 promoting cancer progression [27, 47, 48]. What’s more, it has been reported that stem cells could produce CXCL5, and Zhao et al. demonstrated that CXCL5 secreted by adipose tissue-derived stem cells could promote breast tumor cell proliferation [49]. Thus, we could speculate that CXCL5 might be the indicator of the presence of putative cancer stem cells, which have been shown to be associated with the metastasis and poor prognosis of cancer patients [50, 51].

However, the current meta-analysis had some limitations. First, the cut-off value of high and low CXCL5 expression was different among studies, which might lead to the bias of the results. Second, some HRs could not be directly obtained from the publications. Thus, calculating them through survival curves might not be precise enough. Third, differences of paper quality and sample size across the studies might cause bias in the meta-analysis, although meta-regression did not show the paper quality or sample size as the resource of heterogeneity. Therefore, larger-scale, multicenter, and high-quality studies are desperately necessary to confirm our findings.

## Conclusions

In conclusion, our study revealed that elevated expression level of CXCL5 might be an adverse prognostic marker for OS, PFS and RFS in cancer patients. However, no significant association was found between CXCL5 expression level with DFS in the current meta-analysis. In a word, this is the first meta-analysis to evaluate the relationship between expression levels of CXCL5 with prognosis of cancer patients. In the future, more relevant studies are warranted to investigate the role of CXCL5 in human cancer.

## Abbreviations

CI: confidence intervals
HR: hazard ratios
OS: overall survival
DFS: disease-free survival
PFS: progression-free survival
RFS: recurrence-free survival
IHC: immunohistochemistry
RT-PCR: real time polymerase chain reaction
ELISA: enzyme-linked immunosorbent assay
